# Lived experiences of women with spontaneous abortion at a district hospital, South Africa

**DOI:** 10.4102/safp.v66i1.5917

**Published:** 2024-04-30

**Authors:** Marshall Lockett, Robert J. Mash

**Affiliations:** 1 Division of Family Medicine and Primary Care, Faculty of Medicine and Health Sciences, Stellenbosch University, Cape Town, South Africa

**Keywords:** abortion, miscarriage, pregnancy, patient satisfaction, quality of care, person-centredness

## Abstract

**Background:**

Spontaneous abortions occur in 12.5% of pregnancies and have a significant impact on the well-being of women. Dissatisfaction with health services is well-documented, but no studies have been conducted in district health services of the Western Cape. The aim was to explore the lived experiences of women presenting with spontaneous abortions to the emergency department at Helderberg Hospital.

**Methods:**

A descriptive phenomenological qualitative study used criterion-based purposive sampling to identify suitable participants. Data were collected through semi-structured individual interviews. Atlas-ti (version 22) software assisted with data analysis using the framework method.

**Results:**

A total of nine participants were interviewed. There were four main themes: a supportive environment, staff attitudes and behaviour, the impact of time, and sharing of information. The comfort, cleanliness and privacy of the environment were important. COVID-19 had also impacted on this. Showing interest, demonstrating empathy and being nonjudgemental were important, as well as the waiting time for definitive treatment and the time needed to assimilate and accept the diagnosis. In addition, the ability to give relevant information, explain the diagnosis and help patients share in decision-making were key issues.

**Conclusion:**

This study highlighted the need for a more person-centred approach and managers should focus on changes to organisational culture through training and clinical governance activities. Attention should be paid to the physical environment, availability of patient information materials and sequential coordination of care with primary care services.

**Contribution:**

This study identifies issues that can improve person-centredness and women’s satisfaction with care for spontaneous abortion.

## Introduction

Spontaneous abortion is defined as pregnancy loss before 24 weeks of gestation and can be classified as inevitable, incomplete or complete.^1^ Global estimates suggest that spontaneous abortions are the most common complication of pregnancy and almost 25% of pregnant women will suffer from vaginal bleeding during the first two trimesters, of whom 50% progress to spontaneous abortion.^2^

The experience of a spontaneous abortion is a significant life changing event for women, and dissatisfaction among these women regarding the healthcare received is well-documented.^3,4,5^ Women are mostly disappointed with long waiting times, inadequate information, poor communication, lack of empathy, insensitivity and limited choice regarding the management options.^6,7,8^

In South Africa, the emergency centres of district hospitals serve as the entry point to health services for the majority of patients.^9^ Healthcare interventions focus primarily on medical or surgical management of patients and may neglect to provide adequate psychological support.^3,10^ However, spontaneous abortions are associated with significant mental health consequences, affect quality of life and can impact on future pregnancies.^11^ Furthermore, factors such as lower socioeconomic background, preexisting psychiatric illness and poor social support increase the risk of severe psychological distress in women following a spontaneous abortion.^12,13^ It is estimated that, following a spontaneous abortion, 50% of women suffer from conditions such as anxiety, depression, post-traumatic stress, prolonged grief or guilt.^14,15^ Care in the emergency department though is often the only opportunity for women to receive psychological support.^16,17^ The psychological needs of women should be addressed more effectively, in order to improve care for spontaneous abortions.^4,18^

According to the World Health Organization (WHO), the quality of healthcare is informed by patient experiences and integral to this is person centredness, which is one of the six key dimensions of quality care.^19^ The Western Cape Health Care Plan for 2030 also identified person-centred care as an essential component in improving the experience of patients.^20^ Person-centred care is respectful to individual needs and values.^4,19^ Furthermore, person-centred care encourages the participation of the patient and their family in more shared clinical decision-making.^21^ Moreover, person-centred care emphasises health service delivery that incorporates the patient’s perspective and advocates for the holistic management of the patient in order to improve health outcomes.^19^ Therefore, each patient requires an individualised approach to spontaneous abortion care.^3^

The level of patient satisfaction is an important indicator of the quality of healthcare that services provided.^19^ Both the healthcare workers and the healthcare environment are central to ensuring the delivery of person-centred care and improving patient satisfaction.^22^ Being more person-centred within the emergency department can have a positive impact in reducing the adverse effects of spontaneous abortions.^5,6,23^

A systematic review published in 2022 highlighted the beneficial impact of providing psychosocial support, utilising protocols and effective patient education, on the physical and mental well-being of these patients within emergency departments.^24^ A similar study, focussing on person-centred care, identified 24 different aspects of care, which were associated with improved quality of care and informed intervention strategies to advance spontaneous abortion care.^3^ However, there is a lack of local data on patients’ perspectives of their healthcare in the management of spontaneous abortions within the emergency department setting. The aim of this study was to explore the experiences of healthcare received by women presenting with spontaneous abortions to the emergency department at Helderberg District Hospital in the Western Cape, South Africa. The objectives of this study were to explore the women’s experience of the healthcare workers, the facility infrastructure and environment, and the treatment received during their visit.

## Research methods and design

### Study design

This was a descriptive phenomenological qualitative study using semi-structured interviews. Phenomenological studies explore peoples’ lived experiences and in this case the experience of women seeking care for a spontaneous abortion at a district hospital. This is more of a descriptive study as it attempts to understand what women experienced rather than how they ascribed deeper meaning to this experience.

### Study setting

This was a single centre study conducted at Helderberg Hospital, which is a public sector district hospital, situated in the Cape Town Metropolitan District of the Western Cape Province in South Africa. The Cape Metropole is divided into four substructures and eight subdistricts. Helderberg Hospital is an 181-bed hospital providing 24-h emergency services. It is equipped with two operating theatres and provides outpatient and inpatient services. It serves as a referral centre for the management of suspected spontaneous abortions. Most patients are referred to the hospital’s emergency department from nine local primary care clinics.

The Eastern subdistrict is one of the most densely populated and poorest subdistricts, having more households without income than the rest of the Cape Town Metropole. This catchment area consists of urban and informal settlements, with diverse communities of different ethnic groups. English, isiXhosa and Afrikaans are the main languages spoken. The population is predominantly living in poor socioeconomic circumstances and mainly utilises government emergency medical services and public transport to access the hospital.

At this facility, patients present to reception to obtain a hospital folder and are then directed to a designated area for triage by nursing staff. Patients, who are stable, wait to see a doctor in one of four consultation rooms. The emergency department is equipped with a portable ultrasound unit to assist with diagnosis and management of spontaneous abortions. Patients are then either treated medically and discharged, with a follow-up appointment, or admitted for surgical management.

### Selection of the participants

The study population was adult women (> 18 years) with a diagnosis of spontaneous abortion. Women requesting termination of pregnancy and those with septic abortions or ectopic pregnancies were excluded. Women with acute mental illness were also excluded.

Criterion-based purposive sampling was used to identify suitable participants, particularly women of different parities, different age groups between 18 and 40 years, and from different socioeconomic backgrounds.

The emergency department register was checked for the period December 2020 – April 2021 for patients with either vaginal bleeding or abortions. A total of 183 folders were identified and further checked to specifically identify all patients managed for spontaneous abortions.

The researcher aimed to conduct 8–12 interviews; however, the final sample size was determined by data saturation. Data saturation was reached when no new information was reached from the last two interviews.^25^

### Data collection

An interview guide was developed for the interviews and explored women’s experiences of spontaneous abortion, staff attitudes, hospital infrastructure, the doctor-patient consultation and the psychological impact of the experience. The guide was developed from topics identified in previous qualitative studies.^3,4,5,6,7^

Potential participants were contacted telephonically, at least 6 months after the spontaneous abortion, and invited to participate in face-to-face interviews. This timeframe allowed for emotional space and reduced the possibility of adding to their emotional trauma.

Participants were requested to complete a consent form and provide basic demographic information. The mental health status of each participant was screened using a validated mental health screening tool.^26^ This tool assisted the researcher to identify the risk of mental health disorders. Participants who screened positive for mental health disorders were excluded from further participation and assisted to access mental health services. A registered psychologist was available to support participants suffering acute emotional distress or those in need of counselling.

The semi-structured interviews were conducted in mutually agreed private and safe spaces, between July and October 2021. Strict coronavirus precautions were adhered to. Interviews were audio-recorded and lasted between 30 and 60 min. Interviews were conducted in English or Afrikaans. Field notes were also taken during the interviews. The researcher conducted the interviews and was trained in person-centred communication skills and qualitative interviewing by Stellenbosch University.

### Data analysis

The audio recordings were transcribed verbatim by an independent transcriber. All the transcripts were checked against the audio recordings for accuracy. The framework method of qualitative analysis was used as it is an inductive approach to thematic analysis.^25^ The researcher conducted the analysis with the help of Atlas-ti (version 22) software. The process had the following steps:

- Familiarisation with the data: The researcher familiarised himself with the transcripts and underlying audio data as well as the field notes. He identified issues in the data that could be coded.
- Development of a coding index: Based on step 1, a list of clearly defined codes was developed, and codes were organised into categories.
- Coding: The codes were then used to code the data in all transcripts using Atlas-ti and if necessary new codes were added.
- Charting: The data linked to codes and categories were brought together in report forms generated by Atlas-ti.
- Interpretation: The reports were interpreted for themes and subthemes and any potential relationships between themes.

The analysis process was supervised and reviewed, particularly at steps 2 and 5.

### Trustworthiness

The researcher was a male family medicine registrar working at Helderberg Hospital who had experience of managing women in the emergency department with spontaneous abortions. The researcher practises patient-centred care and has experience with managing the psychological effects of similar women following their encounters at emergency departments. He has worked as a general practitioner in both private practice and public health facilities. The researcher remained aware of his own beliefs and perceptions during data collection and analysis, so that these did not unduly influence the process. Furthermore, the researcher was cognisant that being male, a medical doctor and working at Helderberg Hospital could influence rapport and openness with participants. The researcher fully disclosed his role at Helderberg Hospital and ensured that he was not personally involved in the care of any participants. The researcher was mindful of the need for continuous critical self-reflection during the interviews and analysis phase to limit the impact of potential bias, gender and power dynamics, as both doctor and researcher.

Apart from attention to reflexivity, the work was supervised, particularly the quality of qualitative interviewing, construction of the coding index and interpretation of the data. Atlas-ti enabled a clear audit trail for the analysis process.

## Results

In total, 182 folders were screened for eligibility, of which 141 patients met the criteria for the study and were contacted telephonically. More than 85% of these potential participants were either untraceable or declined to participate. Seventeen participants were both contactable and willing to be interviewed. The final sample size was determined by data saturation, which was reached after nine interviews. Participants came from five different communities in the catchment area. Seven interviews were in English and two in Afrikaans. None of the participants screened positive for mental health conditions or required urgent emotional support during the interviews. The characteristics of the participants are summarised in Table 1.

**TABLE 1:** Sociodemographic characteristics of participants.

Participant	Age (years)	Parity	Education	Marital status
1	23	G1P0M1	Matric	Single
2	33	G3P1M2	Matric	Long-term partner
3	36	G2P1M1	Matric	Married
4	39	G3P2M1	Matric	Long-term partner
5	30	G5P1M4	Matric	Long-term partner
6	38	G3P1M2	Matric	Married
7	27	G1P0M1	Post- matric	Married
8	30	G4P0M4	Matric	Long-term partner
9	39	G3P2M1	Post- matric	Married

Note: G (gravidity) = total number of confirmed pregnancies; P (parity) = total number of pregnancies carried over the threshold of viability; M (miscarriage) = total number of spontaneous abortions.

Most of the participants were in their third decade, and all participants had completed matric. Most participants were employed, and eight were married or in long-term relationships. Five of the participants had experienced one spontaneous abortion; two participants had experienced two spontaneous abortions and two participants had experienced four spontaneous abortions.

Four main themes were identified, namely a supportive environment, staff attitudes and behaviour, the effect of time, and sharing of information. A further nine subthemes arose from the main themes, which are described in Table 2. Information sharing had no subthemes.

**TABLE 2:** Overview of themes and subthemes.

Themes	Subthemes
1. Supportive environment	1.1. Comfort 1.2. Cleanliness 1.3. Privacy 1.4. Effects of COVID-19
2. Staff attitudes and behaviour	2.1. Showing empathy 2.2. Showing interest 2.3. Nonjudgemental
3. Impact of time	3.1. Time waiting from arrival to treatment 3.2. Time to assimilate the diagnosis
4. Information sharing	-

### Theme 1: A supportive environment

Patients commented on the effect of the physical environment, the need for adequate support from healthcare staff, the comforting presence of escorts and how the COVID-19 pandemic impacted their care experience.

#### Subtheme 1.1: Comfort

Most participants criticised the physical condition of the emergency centre and its amenities. Complaints related mostly to the time spent sitting in chairs while being in pain and actively bleeding. Some expressed their dismay at having to wait in an outside waiting area, which was described as cold and uncomfortable:

‘Yes, it really was not nice because it was my first time and I thought that’s okay, I open a file – but then they told me to wait outside, in the outside waiting area. It is literally outside – that was not a nice experience.’ (P9, G3P2M1)

#### Subtheme 1.2: Cleanliness

In general, the emergency department was perceived to be a clean space. However, several participants felt particularly disgusted at having to use blood-stained bathrooms, but simultaneously offered explanations such as staff shortages or the fact that more trauma-related cases were attended to at that particular time of the day. However, this did not lessen their disapproval of the lack of cleanliness encountered in the bathrooms specifically:

‘The bathroom, it’s a mess, but the time you see it’s in the morning, maybe there is no one there. Maybe the cleaners they clean during the day, maybe at night, maybe there are few or there are no cleaners at night. Only the toilet, the bathroom it was a mess, even the walls.’ (P4, G3P2M1)

Conversely, a few patients came across clean bathroom facilities, reporting clean linen on beds and their appreciation for the provision of sanitary pads:

‘That was in the emergency area. To be honest, I don’t know how many people were there, but it was clean – I can confirm that. I lay down on a clean bed waiting for the sister, where normally the sister came and asked you to wait so that they could put clean linen on the bed.’ (P9, G3P2M1)

#### Subtheme 1.3: Privacy

A lack of privacy during physical examination was another concern raised by patients, who shared their unhappiness with the curtains around the trolley and suggested that they would have preferred a private examination room. They felt uncomfortable knowing that anyone was able to enter the examination space without warning, particularly male porters and security staff. This gave rise to anxiety and patients felt exposed:

‘There was a curtain there, but in the middle of the curtain it doesn’t close, so it’s open there. I don’t know if they were doctors or not, but like the one actually opened the curtain while she was busy. And then she just closed the curtain, but I could still see the opening there in between.’ (P3, G2P1M1)

#### Subtheme 1.4: The effect of COVID-19

According to patients, they encountered a less busy emergency department due to COVID-19, with quicker and improved service delivery. However, the restricted visitor protocols also meant that escorts were not allowed to accompany patients and adhering to social distancing regulations meant an outside waiting area. Patients expressed feeling alone, but none voiced their disagreement with the protocols. Nevertheless, patients emphasised unfairness with the application of the rules, having noticed staff allowing escorts with other patients. Although patients understood and adhered to these policies, the majority sought the presence of a loved one for support during this emotional time:

‘… having had to go through the pain and that whole experience, there is somebody outside who can be there with you to hold your hand and support you. But now you have to go through this alone because the rules are that nobody is allowed to go in, but being on your own, alone during such an experience is not nice.’ (P9, G3P2M1)

One participant expressed her suspicion of receiving inferior treatment due to COVID-19 protocols, which could have negatively impacted her pregnancy. This particular concern gave rise to scepticism and mistrust in the motives of healthcare staff:

‘So, I wondered if they did not want to try and stop it [*miscarriage*], is it because they don’t want to fill the hospital because of the Covid pandemic, so they did not want to admit patients unnecessary because they wanted to keep the bed available for someone else.’ (P9, G3P2M1)

### Theme 2: Staff attitudes and behaviour

#### Subtheme 2.1: Showing empathy

Almost all participants interpreted staff attitudes and behaviour as demonstrating a lack of empathy, concern or compassion. Participants expected to be treated with compassion. Some participants felt that staff were unfriendly, describing them as rude and not showing any emotion:

‘Just be empathetic towards the people that is there. Be considerate of the people that is sitting there, that is sick, that is coming for help. You know, be friendly. At least greet. Good evening, morning, it costs them nothing. How can I help? Not just come, take your blood, test your urine and then you know.’ (P7, G1P0M1)

‘So and while he was busy with me, I didn’t see any emotion on his face like it was just nothing, he was just working on me and seeing what’s the problem and that’s it.’ (P1, G1P0M1)

#### Subtheme 2.2: Showing interest and validating my concerns

Staff were perceived as being dismissive and disinterested, reportedly ignoring participants’ concerns, even when these concerns were communicated explicitly. Patients expected healthcare staff to respond appropriately when expressing their feelings or discomfort. One participant was left feeling unheard as she described how staff accused her of being overdramatic and felt that her concerns were minimised. Patients wanted their concerns to be acknowledged and validated:

‘I’m telling them that something is happening … she [*nurse*] didn’t even say okay come let me check. She said I’m lying that how can the baby come out when I’m not bleeding. And she didn’t even check if I’m bleeding or not.’ (P2, G3P1M2)

#### Subtheme 2.3: Do not judge me

Healthcare staff were perceived as judgmental, and the following quote is an example of how one participant felt judged and criticised for making use of a wheelchair due to their discomfort:

‘They don’t treat you like you are now going to be a mother, or that something happened to you. They don’t treat you like that. And like the nurses they … I don’t know because if you’re young maybe the way they look at you, but I think you can’t choose whether you fall pregnant or not. Different people have different reasons.’ (P3, G2P1M1)

Conversely, when staff displayed concern, validated people’s emotions and showed empathy, the patients felt listened to and comforted. These supportive interactions had a positive influence on the participants’ experiences:

‘So, she made me feel at peace, like maybe felt comfortable and everything and she actually gave me my privacy afterwards coz she could see I was traumatized. So she gave me privacy and everything just to breathe and to think about everything …’ (P1, G1P0M1)

Several participants also mentioned how small gestures by staff had a positive effect on their care experience. This included being remembered by doctors from a previous visit, being friendly, ensuring they were comfortable by providing an extra blanket or holding their hands:

‘The way she talked to me and explained to me what is happening – she told me she was sorry about it. She took my hand and said I’m sorry about what happened to you. She spoke to me and tried to make me feel better – it is the first time that a doctor did that for me.’ (P9, G3P2M1)

### Theme 3: The impact of time

#### Subtheme 3.1: Time waiting from arrival to treatment

Participants’ experience of the time taken to help them and how long they waited varied. Participants were expected to wait at various stations before being consulted by a doctor, receiving medication or being admitted to the hospital. Most participants reported their satisfaction with the short waiting times experienced and being attended to by a doctor in less than an hour:

‘But the best here – at reception it was very, everything was quick. I waited only about half an hour for a doctor, and the surroundings were calm in trauma.’ (P8, G4P0M4)

One participant expressed her amazement at the unexpectedly quick assistance received compared to the time spent at the referral facility:

‘I had less than five minutes to go and see the doctor. That’s why I say at the hospital, I didn’t take long. It was quick like, I didn’t even expect, because when I come from the first one, I felt like I spend the whole day and sleep there. Maybe it’s going to be there again, but I was shocked that it didn’t take that long.’ (P4, G3P2M1)

However, a few participants expressed their dissatisfaction with the long waiting periods and even felt that this might have had a negative impact on the outcome of their pregnancies:

‘And their service was so poor, it was … they were so slow with me. And I just feel like maybe they could be the reason why I got a miscarriage. Because I was sitting and waiting for them to help me.’ (P1, G1P0M1)

The time spent waiting could directly influence a participants’ perception of the standard of healthcare received and negatively impact their well-being if prolonged.

#### Subtheme 3.2: Time to assimilate the diagnosis

The diagnosis of a spontaneous abortion was often unexpected and could result in people feeling numb or shocked. Patients appreciated being given some time to process the initial news, before continuing with the discussion on management options:

‘… when I was done, she asked me should she leave me alone? And I said yes please. And she actually gave me that … respected my decision and she left and after a while she came back.’ (P1, G1P0M1)

### Theme 4: Information sharing

Most participants shared their dissatisfaction at the lack of detailed information received from clinicians. They felt excluded from participating in their care decisions and expressed their desire for information to be delivered with more sensitivity and for explanations to be less ambiguous:

‘But I mean everything, that trauma that goes together with that, that pain, it could have been avoided if I had received the correct information. Because I don’t know how these things work. This was my first miscarriage ever.’ (P7, G1P0M1)

Several patients mentioned that the dialogue with clinicians was unilateral and that treatment decisions were made without their input. The expectation was for healthcare staff to be more attentive, allow patients the opportunity to ask questions, provide adequate feedback and value their involvement in discussing treatment options:

‘No, there was no choice. They said that I need to take this medication and also to my understanding you need to obviously clean your inside because if there’s old blood or whatever sitting there or that didn’t come out, it could affect you in the long run you know.’ (P6, G3P1M2)

Some patients were left confused because they had no clear understanding of the cause of their spontaneous abortion, adding to their emotional distress, even after leaving the hospital. Similarly, others felt anxious and unprepared for what to expect in the days following their visit. This led to ambivalence, with patients doubting the prescribed treatment, and concerned that their own actions might be the cause for the spontaneous abortion:

‘She told me “no, we don’t need to share that information with you and you just need to take this medicine” cause they gave me medication. And then I was like under the impression … people was telling me don’t just take medication, when you don’t know what’s going on, cause sometimes the baby can be saved, cause if they took two blood tests from you there was a possibility that, you know, there will still, because every time they took a pregnancy test, it was still showing positive.’ (P6, G3P1M2)

A few participants positively described how the information shared during the consultation was complimented by more detailed information pamphlets and appreciated support from nursing staff, who could address any uncertainties and reinforce their understanding of the processes involved:

‘After getting dressed I went to the sister who asked me, before she gave me the tablet, she asked me if the doctor explained what it is for and I said yes she did, and I also got a pamphlet with it where the name of the tablet is on, what the pill is for.’ (P9, G3P2M1)

This highlighted the value of having different information sharing modalities to strengthen participants’ understanding of what to expect at different times of their care.

## Discussion

### Summary of key findings

**FIGURE 1:**
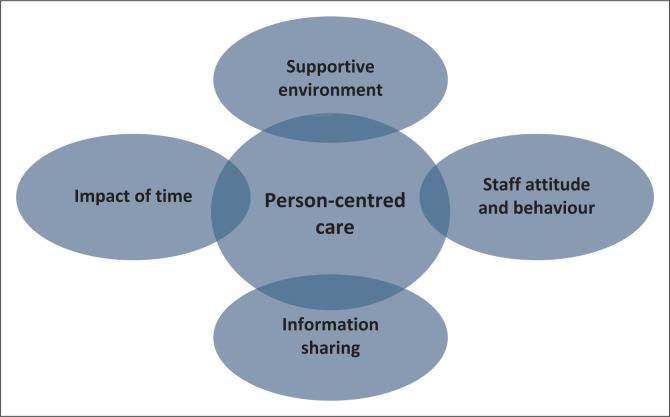
Summary of key findings.

The key findings of the study are illustrated in Figure 1, depicting the four main factors which influenced women’s perception of the healthcare received and the relationship to person-centred care. Person-centred care can have a direct impact on women’s satisfaction with the healthcare services.

### Discussion of key findings

Healthcare facilities and workers are obligated to deliver public services that encapsulate a people-first approach, within the framework of the Batho Pele principles, and which resonate with person-centred care.^20^ Patient dissatisfactions, due to an unfriendly environment, lack of emotional support and poor communication, have been reported on in other studies.^24,27^ Furthermore, our findings mirror systematic reviews on patients’ expectations of spontaneous abortion care, emphasising the desire to be treated as people experiencing a significant life event, rather than just another patient.^3,28^ As noted in this study, patients felt distressed when they were not treated with dignity in a private space, were denied access to emotional support and did not receive adequate information.

The positive responses to waiting times and the disappointment with uncomfortable facilities should be considered in the context of the COVID-19 pandemic. Patient attendance at emergency departments was significantly decreased, as a consequence of the national lockdown measures in South Africa.^29^ A recent study, conducted at a district hospital in Cape Town, reported a 35% reduction in emergency department visits and 56% overall reduction in waiting times for consultation during the COVID-19 pandemic.^30^ The overall satisfaction with short waiting times, reported by the majority of our patients, is contrary to similar qualitative studies, where patients expressed their dissatisfaction with long waiting times.^6,7,24,28^

The COVID-19 pandemic resulted in the implementation of restrictive health measures, such as outdoor waiting areas, to observe strict social distancing and the limitation of visitation privileges, in line with national lockdown policy.^29^ Interestingly, our patients were disgruntled with the inconsistent application of these measures by healthcare staff, particularly when escorts were allowed in with other patients. Similar to our findings, studies have reported on how being separated from your partner and support during the COVID-19 pandemic exacerbated feelings of distress and loneliness in women experiencing pregnancy loss.^31,32^

Staff behaviour and attitudes play a significant role in how patients experience the care process and influence emotional well-being.^12,33^ Several studies have also shown that the lack of empathy and compassion by emergency department staff contributes to dissatisfaction and adverse psychological outcomes, particularly when patients perceived a lack of caring that fails to meet their emotional needs.^7,24,33^ Our study supports these findings and highlights how depersonalisation of care may be a normative experience. These indifferent attitudes affected healthcare staff’s ability to recognise and respond appropriately to the emotional concerns of the patients. The emotional impact of pregnancy loss is a shared experience and a systematic review revealed how nurses purposefully limit interaction with patients or keep themselves busy with other tasks, as coping mechanisms.^34,35^ Moreover, a South African study highlighted the negative impact of burnout and secondary traumatic stress on the ability of nurses to deliver compassionate care.^36^ Nurses also mentioned feeling ill-equipped and lacking confidence to provide meaningful emotional support and care, emphasising the need for skilled counsellors.^34,35^ Nonetheless, patients clearly valued acknowledgement of their distress and experienced more compassionate care when healthcare workers respected the emotional impact of their pregnancy loss.

This study also emphasised several areas of concern, which related to poor communication and information sharing. Communication skills, such as breaking bad news, are common and often challenging to healthcare workers due to insufficient training and lack of experience.^37,38^ The poor timing of explanations, the lack of shared decision-making and the failure to provide adequate information contributed to feelings of ambiguity and scepticism regarding the overall management process. Patients remarked on their unhappiness when doctors lacked the time to fully involve them in the management plan and did not take sufficient time to explain the care process to them. Another significant finding was that patients appreciated being allowed the space and time alone, to assimilate the diagnosis. Poor communication skills, coupled with inexperience, can result in failure to adequately address the information needs of patients with pregnancy loss.^39^ Studies from the United States, the United Kingdom and New Zealand have shown marked improvement in patient satisfaction when evidence-based counselling and shared decision-making formed part of the care process, positively affecting their ability to recall information shared.^23,40,41^ Our findings support the importance of improving counselling and communication skills throughout the spontaneous abortion process.

### Strengths and limitations

This was a small study, which was conducted at a single district hospital. It is therefore possible that the views of these participants might not accurately represent the views of all patients that present with spontaneous abortion to the emergency departments in the subdistrict. However, participant diversity and the representation of all the communities within the drainage area enabled this study to gain sufficient in-depth perspectives from nine participants to reach data saturation. In addition, the in-person interviews provided rich data which incorporated participants’ emotions and expressions. These factors increase the credibility and transferability of our findings to emergency departments with similar settings.

### Implications

There is an opportunity to encourage the adoption of a more person-centred culture within the emergency department, which will require commitment from all levels of personnel to effect organisational culture change within a supportive policy framework.^42,43^ This can be achieved by implementing strategies such as in-service training focussed on updating staff on person-centred communication skills and professional development which should be incorporated as clinical governance activities.^44,45^

Management should ensure clean amenities and dedicated private consultation rooms in order to create a hygienic environment and safe space for patients to process their experiences. The findings of this study can guide facility managers to implement tailored strategies, such as workshops on professional values and ethics of care, to develop positive interpersonal behaviour. Targeted person-centred communication skills training should emphasise that a biopsychosocial approach with a guiding style to counselling will improve skills related to breaking bad news and shared decision-making. This will ensure that immediate supportive counselling can be offered by doctors and nursing staff within the emergency department. Moreover, the availability of comprehensive information pamphlets with what to expect and the reinforcement of the care plan by other healthcare staff were associated with improved understanding and satisfaction among our patients. Follow-up plans should be clear and ensure easy access to supportive aftercare. This can be improved through re-evaluating down-referral pathways to community health centres and partnering with appropriate non-profit government organisations and community structures to provide ongoing counselling and support.

Furthermore, future research should include the experiences of patients attending private hospitals and explore the viewpoints of healthcare staff working in the emergency departments, as well as family members and escorts, to obtain a more holistic perspective on person-centred care practices within the emergency department.

## Conclusion

The findings of this study indicate the need for improvement of the care process for women experiencing spontaneous abortion in the emergency department and it is evident that healthcare workers and managers can positively influence the patient’s care experience. Implementing practical interventions to advance person-centred care can contribute towards meeting the patient’s expectations of being managed in a supportive environment, by empathetic healthcare staff who include them in decision-making while providing comprehensive information.
